# Variability of DNA Microarray Gene Expression Profiles in Cultured Rat Primary Hepatocytes

**Published:** 2007-11-18

**Authors:** Jun Xu, Xutao Deng, Victor Chan, Nancy Kelley-Loughnane, Brent W Harker, Leming Shi, Saber M. Hussain, John M. Frazier, Charles Wang

**Affiliations:** 1 Department of Medicine and Burns and Allen Research Institute, Cedars-Sinai Medical Center, Los Angeles, CA 90048; 2 David Geffen School of Medicine at UCLA, Los Angeles, CA 90048; 3 Alion Science and Technology, Inc., Dayton, OH, 45433; 4 Geo-Centers, Inc., WPAFB, OH 45433; 5 Center for Tropical Disease Research and Training, University of Notre Dame, Notre Dame, IN 46556; 6 National Center for Toxicological Research, U.S. FDA, Jefferson, AR 72079; 7 U.S. Air Force Research Laboratory, WPAFB, OH 45433

**Keywords:** microarray, variability, sample size, hepatocytes

## Abstract

DNA microarray is a powerful tool in biomedical research. However, transcriptomic profiling using DNA microarray is subject to many variations including biological variability. To evaluate the different sources of variation in mRNA gene expression profiles, gene expression profiles were monitored using the Affymetrix RatTox U34 arrays in cultured primary hepatocytes derived from six rats over a 26 hour period at 6 time points (0h, 2h, 5h, 8h, 14h and 26h) with two replicate arrays at each time point for each animal. In addition, the impact of sample size on the variability of differentially expressed gene lists and the consistency of biological responses were also investigated. Excellent intra-animal reproducibility was obtained at all time points with 0 out of 370 present probe sets across all time points showing significant difference between the 2 replicate arrays (3-way ANOVA, *p* ≤ 0.0001). However, large inter-animal biological variation in mRNA expression profiles was observed with 337 out of 370 present probe sets showing significant differences among 6 animals (3-way ANOVA, *p* ≤ 0.05). Principal Component Analysis (PCA) revealed that time effect (PC1) in this data set accounted for 47.4% of total variance indicating the dynamics of transcriptomics. The second and third largest effects came from animal difference, which accounted for 16.9% (PC2 and PC3) of the total variance. The reproducibility of gene lists and their functional classification was declined considerably when the sample size was decreased. Overall, our results strongly support that there is significant inter-animal variability in the time-course gene expression profiles, which is a confounding factor that must be carefully evaluated to correctly interpret microarray gene expression studies. The consistency of the gene lists and their biological functional classification are also sensitive to sample size with the reproducibility decreasing considerably under small sample size.

## Introduction

DNA microarray is one of the most powerful tools that allows the measurement of thousands of genes simultaneously and has been used extensively in biomedical researches [1–9]. Like all other biological studies, variations in DNA microarray experiments are inevitable and DNA microarray experiments can be affected by numerous nuisance variables including experimental design, sample preparation and chip process and others [10–12]. Generally speaking, there are two major sources of variations involved in microarray experiments: technical variation and biological variation [11; 13]. Technical variations may occur during microarray chip manufacturing and/or sample processing including RNA extraction and purification, cDNA synthesis, *in vitro* transcription, chip hybridization, staining, washing and chip scanning (measurement error) [12]. Biological variation is the intrinsic differences of gene expression profiles among individuals in nature due to genetic and/or environmental factors [11; 12; 14; 15]. Although the technical reproducibility across different labs and different platforms have been carefully studied [14–18], the issue of biological variability of gene expression profiling, particularly in time-course expression profiling studies has not been fully addressed. Some publications have reported that large inter-animal biological variation exist in gene expression profiles [19–26]. A common practice to overcome the biological variation is to estimate the sample size necessary to reach certain statistical power based on the results from a pilot study. However, due to the relatively high cost of microarray experiments, it is not practical to follow such a procedure in gene expression profiling experiments using microarray technique. Thereby, a better understanding of the variability derived from the biological replicates and the effects of sample size on the reproducibility of gene lists are critical to draw a meaningful conclusion from microarray experiments.

In this paper, both inter-animal variation (biological variation) and intra-animal variation (technical variation) were studied using a time-course gene expression data set generated from the primary rat hepatocytes derived from six rats using the Affymetrix Rat Toxicology U34 arrays. This microarray data set was uniquely suitable for the evaluation of the variability of gene expression. Firstly, the cultured primary rat hepatocytes are a very valuable tool and have been widely used for testing toxicological and pharmacological effects of chemicals and drugs [27–29]. Secondly, the study was comprised of both biological replicates (6 animals) and technical replicates (two arrays at each time point/animal), which allowed us to evaluate these two major variations simultaneously. In addition, the technical replicates used in this study were not simply replicates of measurements of the same RNA sample, instead the replications started from the independent culture of hepatocytes derived from the same animal. Lastly, this was a time-course transcriptomic profiling study that allowed one to evaluate the gene expression variations across different time points. Our study demonstrated that an excellent technical reproducibility of gene expression profiling using microarray technology could be obtained. However, biological variability did exist in the animal study and it accounted for a substantial portion of the total variation observed. In addition, our study using both fold-ranking and gene ontology methods showed that the sample size is a critical factor in identifying consistent differentially expressed gene lists from a microarray study.

## Methods

### Chemicals and reagents

Collagenase was obtained from Boehringer-Mannheim Biochemicals (Indianapolis, IN). 3-(4, 5-Dimethylthiazol-2-yl)-2, 5-diphenyltetrazolium bromide (MTT), β-nicotinamide-adenine dinucle-otide-reduced (NADH), insulin/transferrin/sodium selenite (ITS) additive, gentamicin, dexamethasone, dithiothreitol (DTT), ethylenediaminetetraacetic acid (EDTA), phenylmethanesulfonyl fluoride (PMSF), ethylene glycol-bis(2-aminoethylether)-N,N,N′,N′-tetraacetic acid (EGTA), and 4-(2-hydroxyethyl)piperazine-1-ethanesulfonic acid (HEPES) were purchased from Sigma Chemical Company (St. Louis, MO). Chee media was obtained from Gibco (Grand Island, NY). Qiagen RNeasy mini kits were purchased from Qiagen (Valencia, CA). The SuperScript Choice system was purchased from Invitrogen (Rockville, MD) and oligo-(dT) 24 anchored T7 primer was purchased from Amersham (Amersham Pharmacia Biotech, Piscataway, NJ). BioArray high yield RNA transcript labeling kit was purchased from Enzo (Enzo Diagnostics, Inc., Farmingdale, NY). Streptavidin-phycoerythrin was purchased from Molecular Probes (Eugen, OR). Biotinylated anti-streptavidin was obtained from Vector Laboratories (Burlingame, CA).

### Animals

Six male Fischer 344 rats were purchased from Charles River Laboratory (Raleigh, NC). They were housed in a climate-controlled (21 °C) room under a 12-h light–dark cycle and were given tap water and Rodent Chow 5001 (Ralston Purina, St. Louis, MO) *ad libitum.* Rats were anesthetized with 1 ml/kg of a mixture of ketamine (70 mg/ml; Parke-Davis, Morris Plains, NJ) and xylazine (6 mg/ml; Mobay Corp., Shawnee, KS) prior to undergoing liver perfusion. All animals used in this study were handled in accordance with the principles and guidelines prepared by the National Institutes of Health, U.S.A.

### Hepatocyte isolation and culture

A standard protocol was used for the rat liver isolation and culture. Rats were sacrificed at the age around 63 days (250 g–300 g of body weight). Rat livers were perfused, and hepatocytes were isolated and enriched by a two-step Seglen procedure [30] with minor modifications as previously described [28]. Hepatocytes were cultured for 0, 2, 5, 8, 14, 26 hours and mRNA expression was monitored at each time point using Affymetrix RT U34 arrays. Concurrent with cell harvesting for RNA extraction, hepatocytes were collected and assayed for LDH leakage and MTT activity to assess the viability of cell preparations.

### DNA microarray

Affymetrix oligonucleotide arrays (Rat Toxicology U34 GeneChips) containing probe sets interrogating more than 850 genes were used for mRNA expression profiling. DNA microarray analysis was performed according to a procedure as described previously with certain modification [31].

### Data analysis and statistical tools

The intensity for each feature of the arrays was captured with Affymetrix Microarray Suite (MAS 5.0) according to the standard Affymetrix procedures. An average expression signal for each gene was derived from the 20 pairs of probe sets (perfect match vs. mismatch). All chip data were first evaluated according to the Affymetrix Genechip quality control standards. Data from three chips did not meet the necessary QC requirements thereby were excluded. Actually, these three bad chips were due to either chip defects or the problem with hybridization. Data generated from MAS were analyzed by GeneSpring 7 (Silicon Genetics, Redwood City, CA). GeneSpring 7 was also used for GO (Gene Ontology) terms acquisition and corresponding *p*-value computing. Gene expression signal measurements less than 0.01 were set to 0.01. Each gene in a chip was normalized to the median value of all genes in the same chip and then normalized across samples. For statistical analyses, gene expression signals were logarithmic transformed before analyses. Principal Component Analysis was performed using JMP IN 5.1 (Cary, NC) and three-way ANOVA was conducted using the Partek software (St. Charles, MO). C++ programs were developed for performing gene list reproducibility analyses.

## Results

### Intra-animal reproducibility of gene expression

To evaluate intra-animal reproducibility (mainly technical variation), two cRNA samples (5 μg each) prepared from two parallel RNA samples derived from the same animal and hepatocyte isolation were hybridized respectively to two chips. The reproducibility between two replicate samples was evaluated using correlation coefficient of signals from all probe sets on the chip. It was found that gene expression profiles of two replicate samples at all time points for all six animals were highly correlated to each other with correlation coefficients (*r*) ranging from 0.922 to 0.990 (Supplemental Materials, Table S1, highlighted in bold, italic font). Three-way ANOVA demonstrated that 0 out of 370 probe sets (all probe sets with “present” calls) were statistically different between the two replicate arrays (Supplemental Materials, Table S2). Hierarchical cluster analysis (average linkage algorithm) using 370 probe sets that were present or marginally present in all samples across all time points also showed that replicate pairs of arrays had the closest distance for the same animal at each time point, and they were clustered side-by-side (Supplemental Materials, Figure S1).

### Inter-animal correlation coefficients of gene expression profiles and inter-animal variation determined by three-way ANOVA

**Figure 1:**
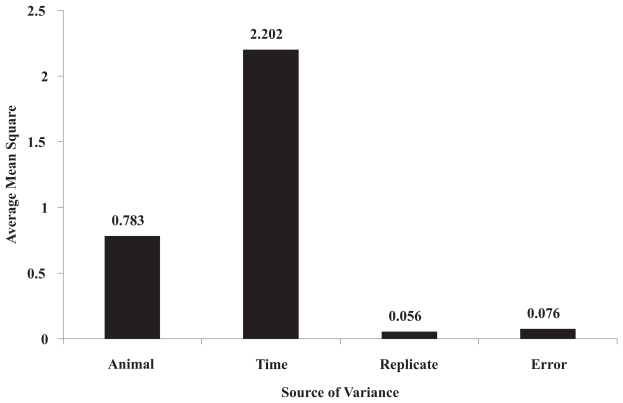
Sources of variation estimated by three-way analysis of variance. Three-way ANOVA was conducted on 370 probe sets that were present or marginally present in all samples across all time points. The number on each bar represents the average of mean square of each variable.

The correlation coefficients of gene expression profiles among the 6 animals ranged from 0.699 to 0.961. As shown in Table S1, the correlation coefficients of gene expression profiles between animal A and the other five animals (B, C, D, E and F) were much less than those among animals B, C, D, E and F (0.857–0.912 vs. 0.894–0.955 at 0 hour; 0.784–0.900 vs. 0.826–0.955 at 2 hours; 0.750–0.908 vs. 0.790–0.955 at 5 hours; 0.840–0.888 vs. 0.895–0.961 at 8 hours; 0.699–0.832 vs. 0.810–0.955 at 14 hours; 0.686–0.875 vs. 0.897–0.959 at 26 hours, respectively), indicating that animals B, C, D, E and F were somehow more closely correlated to each other, whereas animal A seemed to be different from the others in its gene expression profile. In addition, three-way ANOVA results indicated that animal variation was the second largest source of variations (Fig. 1). There were 337 out of 370 probe sets (all probe sets with “present” calls) showing statistical differences among animals (*p* ≤ 0.05), whereas 0 out of 370 probe sets had significant differences between the technical replicate arrays (three-way ANOVA, Table S2). Pairwise comparisons (three-way ANOVA) indicated that there were more genes with significant differences between animal A and other animals (B, C, D, E and F) than those with differences among animals B, C, D, E and F (Supplemental Materials, Table S3). These results indicated that significant inter-animal biological variation of gene expression existed among different animals and that the magnitude of inter-animal variation is much greater than that of intra-animal variation.

### Principal component analysis (PCA)

PCA is a mathematical technique to project the observations (samples) from the high-dimensional variables (genes) space to a low-dimensional subspace spanned by several linear combinations derived from the original variables (genes) to account for the maximum variability in a data set [32; 33]. PCA has been widely used to analyze and visualize multidimensional data sets [34–36].

**Figure 2:**
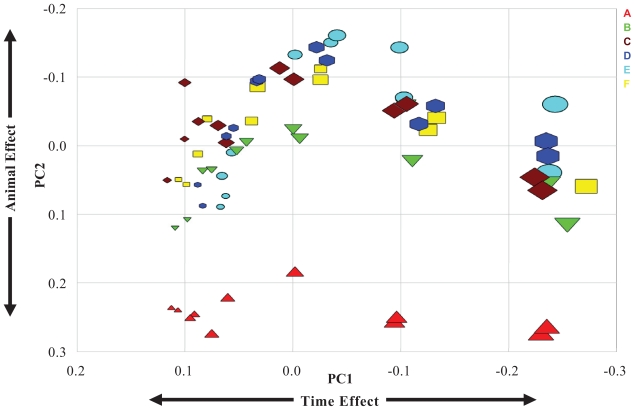
Principal Component Analysis: PC_1_–PC_2_ plane. PCA was performed using 370 probe sets present or marginal present in all six animals. Time points are represented by different symbol size with the smallest size (far left) represent the earliest time point and the largest size represent the latest time point (right). The samples from different animals are represented in different colors: animal A in red, animal B in green, animal C in brown, animal D in blue, animal E in cyan and animal F in yellow.

PCA was applied to examine the sources of variation in the time-course gene expression in rat primary hepatocytes obtained from 6 animals. In total, 370 probe sets (genes) with “present” or “marginally present” calls by MAS 5.0 in all 69 samples (arrays) at all six time points were subject to PCA. Three major principal components were identified and they accounted for 47.4% (PC1), 9.7% (PC2) and 7.2% (PC3) of total variance. These results from the PCA analysis clearly demonstrated that the patterns of mRNA expression were influenced by multiple factors. As shown in Figure 2, in accordance with three-way ANOVA result, PC1 characterized the time effect on the variability of gene expression for each animal (represented by different colors) and it accounted for the major variation of all variance. It was clearly shown that all samples from animals B, C, D, E and F were clustered together at all time points (represented by different symbol sizes), whereas the two replicate samples (arrays) from animal A differed significantly from the other five animals at all time points by PC2. When plotting the data in the PC2–PC3 plane (Supplemental Materials, Figure. S2), significant distances were observed between the clusters of animal A and animal B, C, D, E and F. Hence, PC2 and PC3 reflected the animal effect on the variability of gene expression.

### Numbers of genes (probe sets) with significant differences among animals

In order to identify the numbers of genes that were differentially expressed among animals, one-way ANOVA was performed on the genes that were “present” or “marginally present” at each time point. Table S4 in Supplemental Materials shows the number of probe sets (genes) that were statistically different among the 6 animals at each time point. Of the genes “present” or “marginally present” at each time point, many were found statistically differentially expressed (Supplemental Materials, Table S4, 0h: 200/473; 2h: 222/431; 5h: 114/430; 8h: 123/440; 14h: 225/467; 26h: 179/440). Consistent with what was found by the PCA and three-way ANOVA analysis, animal A had more genes that were statistically differentially expressed when compared with animals B, C, D, E and F than did animals B, C, D, E and F when compared within themselves at each time point. Furthermore, when using a 2-fold difference as the cutoff, it was found that a significant number of genes (probe sets) were differently expressed among six animals (Supplemental Materials, Table S5, 0h: 473; 2h: 431; 5h: 430; 8h: 440; 14h: 467; 26h: 440). Based on the results from aforementioned correlation, PCA, one-way and three way ANOVA tests, we concluded that animal A was an outlier in this data set and was excluded for the following sample size estimation analysis.

### Impact of sample size on the reproducibility of differentially expressed gene lists and their functional classification

**Figure 3:**
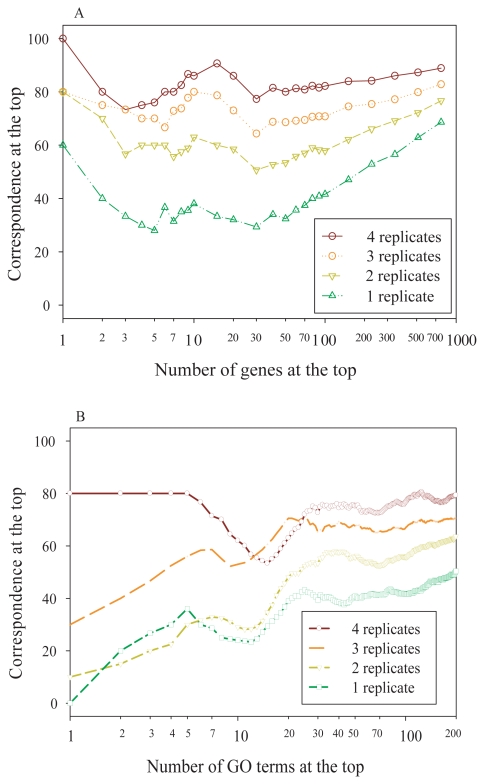
Effect of sample size on the CAT of differentially expressed gene lists and correspondence of enriched GO terms in response to gene lists generated at different sample size. **A.** Differentially expressed genes between 8h and 0h were identified by fold-change using 0h gene expression as reference. The x-axis represents the number of genes selected as differentially expressed from a total of 972 probe sets, and the y-axis represents the overlap of two gene lists. Each curve represents the overlap of a pair of differentially expressed gene lists, one using all replicates and the other using the average derived from a smaller number of replicates in all possible combinations. The comparison was made between the gene lists derived from different sample sizes and the one derived using all animals and the CAT curves are shown as average. **B.** Differentially expressed genes between the 8h and 0h were identified by fold-change using the number of replicates starting from 1 through 5, resulting in 5 gene lists. For each gene list, top 200 genes were selected and were used to derive the rank-ordered enriched GO term lists. Each pair of GO term lists was used to compute the correspondence (y-axis) against the number of GO terms at the top (x-axis), one of the pair using all replicates and the other using a smaller number of replicates. Each CAT curve shows the average of CAT derived from all possible combinations of subset samples for each given sample size (brown, 4 replicates vs 5; orange, 3 replicates vs 5; gold, 2 replicates vs 5; and green, 1 replicate vs 5).

One of the fundamental goals of gene expression profiling experiments is to identify genes that are differentially expressed between the experimental and control groups being studied. Adequate biological replicates are very critical to draw reliable conclusions in microarray experiments. It is known that mRNA expression is very dynamic, displaying different gene expression level across time-course. To illustrate the effect of sample size on the reproducibility of differentially expressed gene lists, we compared differential gene expression across time-course using 0h gene expression as reference. Instead of using the traditional mathematical method to estimate the statistical power and ideal sample size (number of replicates) [37–39] for microarray studies, we focused on the reproducibility of gene lists and the consistency of biological interpretation. *Correspondence at the top* (CAT) graphs [17] were adopted to quantify and visualize the impact of sample size on the differentially expressed gene lists in our data set. A CAT is defined as the percentage of overlapping elements at the top of two ranked lists and CAT graphs were generated by plotting the CAT against the number of elements at the top. An *average CAT*, which was computed by averaging the CAT at 1%, 5%, 10%, 25% and 50% of total elements at the top, was introduced to summarize a CAT graph. Firstly, a reference gene list was generated by ranking expression fold-change between 8h and 0h using all animals (excluding animal A). Then the gene lists using successively decreased sample sizes were generated and compared to evaluate whether reproducible gene lists could be obtained with smaller sample sizes. The CAT graph is shown in Figure 3A, in which CAT was computed by comparing gene lists using different sample sizes against the reference gene list. The average CAT was 84% overlapping with the reference gene list when 4 animals were used and decreased to 75%, 62% and 45% when sample size was reduced to 3, 2 and 1 animal respectively, displaying a 10%–20% average CAT drop when the sample size was decremented. This effect was observed at all pairwise time points comparisons using any subset of animals as replicates (data not shown).

To further evaluate the impact of sample size on the consistency of gene lists based on the biological functional classification, we compared the overlap of gene lists derived from different sample sizes based on their associated Gene Ontology (GO) terms [40]. The top 200 genes based on fold-change were selected from each gene list and subject to GO analysis. Fisher’s exact test was used to calculate *p* value for each GO term associated with these genes. For each given gene list, a GO term was ranked by *p* values with most significant term at the top. CAT graph was then computed by comparing these GO term lists with the reference GO term list generated by using all five replicates (including data from animal B, C, D, E and F). A representative GO term CAT graph comparing differential gene expression between 8h and 0h time points was illustrated in Figure 3B. Similar to what was found using differentially expressed gene lists, the concordance of GO terms was significantly improved in corresponding to the increase of sample sizes.

## Discussion

DNA microarray analysis is a multi-step process, including tissue or cell preparation and treatments, RNA extraction, labeling, hybridization, staining, washing, scanning and data acquisition, and each of these steps could be subject to variations [21; 22; 41; 42]. In order to identify the biologically significant genes differentially expressed across physiological and pathological conditions, it is pivotal to understand the sources of variations of gene expression [19–21; 24; 43; 44]. Huang et al divided these variations into four components: systematic experimental variation, treatment effect, biological variation, and chip variation [42]. However, these variations can be simply classified as technical variability and biological variability [13; 45]. The sources of technical variation of gene expression using GeneChip technology can be controlled and minimized by carefully extracting high quality RNA, standardization of the hybridization, washing, staining, and scanning, as well as the quality control procedures built into manufacturing processes and proper data pre-processing such as scaling and normalization [46–48]. However, the biological variation of gene expression is intrinsic and appears to be at least partially determined by genetic factors [20; 21; 23; 24; 26; 43; 44; 49]. Similar to what has been reported [19; 21], our cultured rat primary hepatocyte gene expression profiling study using Affymetrix RT U34 arrays found that the technical variability is much less significant than the biological variability. We were able to obtain excellent technical reproducibility between two replicate arrays at each time point. The correlation coefficients were found to range from 0.922 to 0.990 for replicate samples (Table S1 in Supplemental Materials) and hierarchical cluster analysis showed that replicate samples had the closest distances and were always clustered side-by-side (Supplement Materials Figure S1). Furthermore, three-way ANOVA analysis demonstrated that none out of the 370 probe sets (all probe sets with present calls) was significantly different between the two replicate arrays (Table S2 in Supplemental Materials), indicating an excellent technical reproducibility. It should be pointed out that, in contrast to what was defined by Yang and Speed [13], our technical replication (intra-animal reproducibility) started from the independent primary hepatocyte cultures derived from the same animal and hepatocyte isolation, which was prior to the RNA extraction step. This level of technical replication actually imbedded certain biological replication, and it seems more challenging and would give much more confidence on the data set if a good reproducibility were obtained.

The biological variability of gene expression using DNA microarray techniques has been investigated by some labs and it was demonstrated that large inter-animal biological variations exist in gene expression profiles [19–26]. It is believed that DNA sequence variation of genes is one of the major biological factors contributing to the phenotypic diversities [20; 21; 23; 24; 26; 43; 44; 49]. Our results also showed a large inter-animal biological variation in gene expression profiles in the rat primary hepatocytes, and this biological variability in gene expression profiles was reflected throughout the time-course studies. The results of using one-way ANOVA, three-way ANOVA and fold-change analysis with a 2-fold difference cutoff as criteria showed that hepatocytes from one animal (animal A) had more genes either statistically different from the other 5 animals (animals B, C, D, E and F) or more genes with at least 2-fold difference compared with other animals (Tables S2–S5 in Supplemental Materials).

To further dissect the sources of variations, PCA was applied to analyze variances of our cultured rat primary hepatocyte gene expression data set. PCA is a statistical technique that allows visualizing the intrinsic relationship of multidimensional data set in a lower dimensional subspace, and it can efficiently illustrate variances of gene expression profiling data [34]. Using PCA, Raychaudhuri et al. demonstrated that much of the variability can be summarized in just a few components capturing most of the information [36]. In our time-course cultured rat primary hepatocyte gene expression profiling data set, it was found that the time effect was the most predominant component of total variances (PC1), and accounted for 47.4% of total variance. This indicates that mRNA expression in cultured rat primary hepatocytes is dynamic and changes with time over the 26h observational period. This observation is consistent with the previous reports that was, in the culture condition, gene expression profiles of primary hepatocytes changed rapidly after isolation with a time-dependent regulation of certain genes including phase I and phase II metabolizing enzymes, cellular cytoskeleton and extracellular matrix genes [29; 50]. In addition, consistent with what was found in three-way ANOVA analysis, there was significant variation between animals (inter-animal variation) with animal effect accounting for 9.7% (PC2) and 7.2% (PC3) of the total variance, respectively. Based on the mean distance between animals at corresponding time points, the difference in gene expression in animal A compared to animals B, C, D, E and F was greater than the difference among B, C, D, E and F, although the replicate samples for all animals were tightly clustered together at all time points (Figs. 2 and S2 in Supplemental Materials). We believe that the larger difference of gene expression profiles between animal A and other rats (B, C, D, E and F) was most likely due to the genetic and biological factors because (1) rat A was fed with the same diet and the liver was harvested at roughly the same age and body weight as for all other animals; (2) an exactly same protocol was used with the hepatocyte isolation and culture as well as for RNA extraction, cRNA labeling, chip hybridization, chip staining and scanning; (3) the hepatocyte viability was evaluated for all animals by both MTT and lactate dehydrogenase leakage assays prior to the mRNA extraction and the cell viability for the animal A was not different from that of other animals (data not shown); 4) QC data for RNA and cRNA, chip hybridization, as well as for the final chip data (background and 3′/5′ ratios of house keeping genes etc) also showed no difference between animal A and the other animals (data not shown).

In order to put our results in context, it is intriguing to compare them with a few recent studies that also investigated sources of variability of gene expression profiling particularly regarding technical variation vs. biological variation [19; 21; 41; 42]. Using unsupervised cluster analysis and correlation coefficients of 92 RNA samples on 76 oligoncleo-tide microarrrays, Bakay et al. reported that experimental error was not a significant source of unwanted variability in expression profiling experiment and the major source of variability was from inter-patient biological variability [19]. Chowers et al. utilized a custom retinal microarray to analyze 33 normal retinas from 19 donors to investigate gene expression variation and they found that a significant fraction of gene expression variation in the normal human retina was attributable to identifiable biological factors [21]. One may argue that the biological variation of gene expression profiles in human samples is definitely larger than that observed in animal samples because so many complex confounding factors contributing to the variations exist in human subjects. However, in animal studies, Huang et al. used measurement of agreement and variance component methods to analyze mouse kidney gene expression profiling data obtained with Affymetrix MG-74Av2 arrays. They found that the biological variability did exist among biological samples, although their analyses indicated that the biological and chip variation were roughly comparable [42]. In other study, Chen et al. applied a linear mixed-effect model to quantify different sources of variation in in-house cDNA array data sets and concluded that the inter-animal variance was smaller than the inter-array variance in four out of five house keeping genes [41]. In spite of these findings, it is hard to obtain convincing results from microarray data set in which the technical variation is greater than the inter-animal biological variation. This view is supported by several recent publications including MicroArray Quality Control (MAQC) studies comparing the reproducibility of microarray gene expression across different labs and different platforms. A high degree of reproducibility both among labs and among platforms could be achieved when standardized protocols were implemented for RNA labeling, hybridization, microarray processing, data acquisition, and data normalization, as well as proper analysis methods utilized [14–18; 51]. Our data clearly indicated that the inter-animal biological variation is larger than the technical variation (intra-animal variability).

Our study also shed lights on the effect of sample size on the reproducibility of differential gene lists that give rise to a consistent biological conclusion. Our results demonstrated that the overlapping gene list declined dramatically when sample size was decreased (Fig. 3A). About 10%–20% average CAT drop was observed when the sample size decreased by 1. A similar decline of overlapping GO terms was also observed.

In summary, our results clearly demonstrate that excellent intra-animal reproducibility can be obtained in the replicate samples of gene expression profiling in cultured rat primary hepatocytes. However, there is large inter-animal variability in the time-course gene expression profiles, which is a confounding factor that must be carefully evaluated to interpret microarray gene expression studies. It is necessary to evaluate the biological variability and identify outliers, if there is any, before any analysis is performed. Furthermore, the biological and technical variability affects the reproducibility of differentially expressed gene lists and the consistency of biological conclusion decreased considerably when the biological replicates were reduced.

## Supplemental Materials

Figure S1 Hierarchical cluster analysis of 69 samples (RatTox U34 GeneChips). Heat map and dendrogram were obtained from 370 probe sets that were present or marginally present in all samples across all time points. Each column represents a sample (chip) and each row represents a gene. The replicate samples for each animal at each time points were clustered side-by-side, indicating a high level of technical consistency of microarray data.

Figure S2 3-D view of principal component analysis: PC1 – PC2 – PC3 projection. PCA was performed on 370 probe sets present or marginally present in all 6 animals. Time points are represented by different symbol sizes. The samples from different animals are represented in different colors: animal A in red, animal B in green, animal C in brown, animal D in blue, animal E in cyan and animal F in yellow.

Table S1 Intra- and inter-animal correlation coefficients (*r*) of gene expression profiles.

**Table:** 

			A		B		C		D		E		F

		Replicate	R1	R2	R1	R2	R1	R2	R1	R2	R1	R2	R1
0h	A	R1											
		R2	***0.978***										
	B	R1	0.883	0.882									
		R2	0.896	0.895	***0.985***								
	C	R1	0.857	0.874	0.936	0.943							
		R2	0.894	0.902	0.939	0.945	***0.967***						
	D	R1	0.895	0.886	0.918	0.916	0.928	0.935					
		R2	0.901	0.904	0.926	0.927	0.935	0.928	***0.983***				
	E	R1	0.888	0.886	0.934	0.938	0.928	0.938	0.905	0.918			
		R2	0.873	0.870	0.930	0.935	0.932	0.928	0.894	0.910	***0.990***		
	F	R1	0.908	0.912	0.926	0.934	0.942	0.951	0.939	0.938	0.955	0.949	
		R2	0.887	0.895	0.916	0.922	0.936	0.932	0.929	0.931	0.944	0.946	***0.982***
2h	A	R1											
		R2	***0.980***										
	B	R1	0.888	0.891									
		R2	0.873	0.881	***0.983***								
	C	R1	0.780	0.784	0.826	0.831							
		R2	0.785	0.797	0.876	0.875	***0.945***						
	D	R1	0.859	0.887	0.927	0.917	0.891	0.932					
		R2	0.860	0.887	0.921	0.913	0.881	0.923	***0.985***				
	E	R1	0.885	0.882	0.951	0.934	0.878	0.915	0.901	0.899			
		R2	0.889	0.892	0.948	0.937	0.877	0.910	0.901	0.896	***0.988***		
	F	R1	0.900	0.898	0.927	0.917	0.893	0.914	0.923	0.916	0.948	0.955	
		R2	0.875	0.887	0.919	0.926	0.884	0.904	0.932	0.932	0.926	0.935	***0.963***
5h	A	R1											
		R2	***0.922***										
	B	R1	0.813	0.872									
		R2	0.800	0.871	***0.964***								
	C	R1	0.811	0.894	0.946	0.955							
		R2	0.750	0.862	0.934	0.952	***0.980***						
	D	R1	0.807	0.875	0.927	0.919	0.927	0.927					
		R2	0.795	0.860	0.918	0.928	0.923	0.923	***0.974***				
	E	R1	0.732	0.764	0.804	0.814	0.790	0.804	0.834	0.829			
		R2	NA	NA	NA	NA	NA	NA	NA	NA	NA		
	F	R1	0.816	0.908	0.934	0.931	0.944	0.942	0.942	0.920	0.801	NA	
		R2	0.757	0.832	0.908	0.901	0.889	0.867	0.895	0.883	0.826	NA	***0.947***
8h	A	R1											
		R2	NA										
	B	R1	0.880	NA									
		R2	0.850	NA	***0.970***								
	C	R1	0.866	NA	0.959	0.961							
		R2	0.858	NA	0.954	0.950	***0.978***						
	D	R1	0.846	NA	0.925	0.930	0.939	0.934					
		R2	0.835	NA	0.910	0.920	0.923	0.916	***0.988***				
	E	R1	0.854	NA	0.939	0.936	0.944	0.943	0.931	0.912			
		R2	0.840	NA	0.929	0.917	0.911	0.930	0.912	0.895	***0.972***		
	F	R1	0.888	NA	0.937	0.929	0.950	0.947	0.932	0.906	0.928	0.929	
		R2	0.853	NA	0.920	0.921	0.930	0.947	0.923	0.906	0.942	0.940	***0.940***
14h	A	R1											
		R2	***0.989***										
	B	R1	0.703	0.699									
		R2	0.802	0.812	***0.972***								
	C	R1	0.750	0.761	0.918	0.927							
		R2	0.737	0.744	0.934	0.955	***0.971***						
	D	R1	0.782	0.793	0.924	0.926	0.928	0.934					
		R2	0.764	0.780	0.914	0.912	0.913	0.921	***0.978***				
	E	R1	0.741	0.749	0.927	0.941	0.923	0.926	0.918	0.918			
		R2	0.803	0.805	0.927	0.950	0.924	0.932	0.930	0.912	***0.980***		
	F	R1	0.728	0.732	0.902	0.903	0.810	0.887	0.871	0.873	0.889	0.910	
		R2	0.832	0.830	0.937	0.947	0.904	0.939	0.927	0.915	0.932	0.946	***0.975***
26h	A	R1											
		R2	***0.970***										
	B	R1	0.787	0.800									
		R2	0.847	0.835	***0.967***								
	C	R1	0.788	0.786	0.930	0.951							
		R2	0.775	0.781	0.942	0.954	***0.984***						
	D	R1	0.856	0.856	0.915	0.928	0.934	0.931					
		R2	0.859	0.866	0.911	0.929	0.933	0.931	***0.981***				
	E	R1	0.824	0.822	0.930	0.937	0.917	0.912	0.907	0.902			
		R2	0.686	0.699	0.908	0.897	0.927	0.923	0.913	0.906	***0.945***		
	F	R1	0.875	0.867	0.928	0.950	0.930	0.934	0.932	0.927	0.943	0.959	
	R2	NA	NA	NA	NA	NA	NA	NA	NA	NA	NA	NA	

1 A, B, C, D, E and F represent six different animals.

2 R1 and R2 are replicate chips from each animal at each time point.

3 Correlation coefficients (*r*) of intra-animal replicate samples are presented in bold italic font.

Table S2 Numbers of probe sets with significant difference determined by three-way analysis of variance.

**Table:** 

Variable	Number of probe sets with significant difference ^1^
Animal ^2^	337
Time ^3^	306
Replicate ^4^	0
Model	352

1 In total, 370 probe sets (genes) present or marginally present in all samples across all time points were analyzed.

2 Microarray data were obtained from six animals (A, B, C, D, E and F).

3 Six time points were studied (0h, 2h, 5h, 8h, 14h and 26h).

4 Two replicates were collected for each animal at each time point.

Table S3 Numbers of probe sets with significant difference as estimated by three-way ANOVA pair-wised comparison between animals.

**Table:** 

Animals ^2^	Number of probe sets with significant difference ^1^
A–B	223
A–C	218
A–D	230
A–E	243
A–F	233
B–C	63
B–D	182
B–E	165
B–F	136
C–D	157
C–E	158
C–F	134
D–E	195
D–F	159
E–F	96

1 In total, 370 probe sets (genes) present or marginally present in all samples across all time points were analyzed.

2 Two replicates of microarray data were obtained from each animal (A, B, C, D, E and F) at each time point (0h, 2h, 5h, 8h, 14h and 26h).

Table S4 Numbers of genes showing statistical difference of gene expression among animals.§

**Table:** 

		A	B	C	D	E
**0h**	B	44				
(200/473) *	C	40	24			
	D	35	68	43		
	E	77	68	61	90	
	F	36	43	27	47	47
**2h**	B	47				
(222/431) *	C	38	39			
	D	61	68	44		
	E	62	87	88	113	
	F	30	45	30	43	47
**5h**	B	50				
(114/430) *	C	34	29			
	D	52	50	41		
	E	63	65	71	59	
	F	29	32	23	32	40
**8h**	B	31				
(123/440) *	C	58	24			
	D	74	40	77		
	E	55	35	47	83	
	F	36	27	35	61	11
**14h**	B	58				
(225/467) *	C	96	21			
	D	93	24	30		
	E	99	37	57	53	
	F	90	37	44	45	57
**26h**	B	71				
(179/440) *	C	77	57			
	D	82	127	116		
	E	81	75	73	95	
	F	62	28	40	60	26

§ One-way ANOVA was conducted to identify probe sets that were differently expressed among six animals at each time point.

* Numbers of probe sets statistically different out of total probe sets either present or marginally present at each time point in all animals are presented in parentheses (p ≤ 0.05).

Table S5 Numbers of genes with two-fold or greater difference in gene expression among animals.§

**Table:** 

		A		B		C		D		E	
		<	>	<	>	<	>	<	>	<	>
**0h**	B	4	21								
(473) *	C	5	16	4	8						
	D	6	17	10	14	8	11				
	E	19	16	14	5	15	5	26	7		
	F	7	13	16	10	9	4	10	9	4	10
**2h**	B	4	17								
(431) *	C	10	35	5	18						
	D	13	19	9	9	15	5				
	E	21	8	19	2	39	5	26	4		
	F	8	11	15	8	21	8	14	7	3	10
**5h**	B	12	19								
(430) *	C	6	17	5	2						
	D	21	23	9	7	8	5				
**8h**	E	50	52	31	34	31	38	19	39		
(440) *	F	31	16	17	1	11	5	7	3	34	17
	B	3	24								
	C	5	29	4	2						
	D	26	29	11	10	6	8				
	E	24	28	16	4	12	3	19	6		
	F	11	15	17	1	4	0	12	4	5	5
**14h**	B	16	17								
(467) *	C	19	23	2	6						
	D	22	15	6	11	8	7				
	E	27	23	11	7	16	2	20	8		
	F	20	23	6	10	15	7	13	15	5	18
**26h**	B	20	24								
(440) *	C	24	16	6	2						
	D	24	13	12	16	6	11				
	E	33	17	7	4	4	6	18	9		
	F	18	13	6	7	8	8	13	13	4	9

§ A fold-change analysis was conducted to identify probe sets that were differently up or down expressed between animals at each time point.

* Numbers of probe sets with a 2-fold or greater difference in gene expression out of total probe sets either present or marginally present at each time point in all animals are presented in parentheses.
