# Supplementary figures and images for: Variability of DNA Microarray Gene Expression Profiles in Cultured Rat Primary Hepatocytes
Source: Gene Regul Syst Bio. 2007 Nov 18;1:235–49. (PMC2759134)

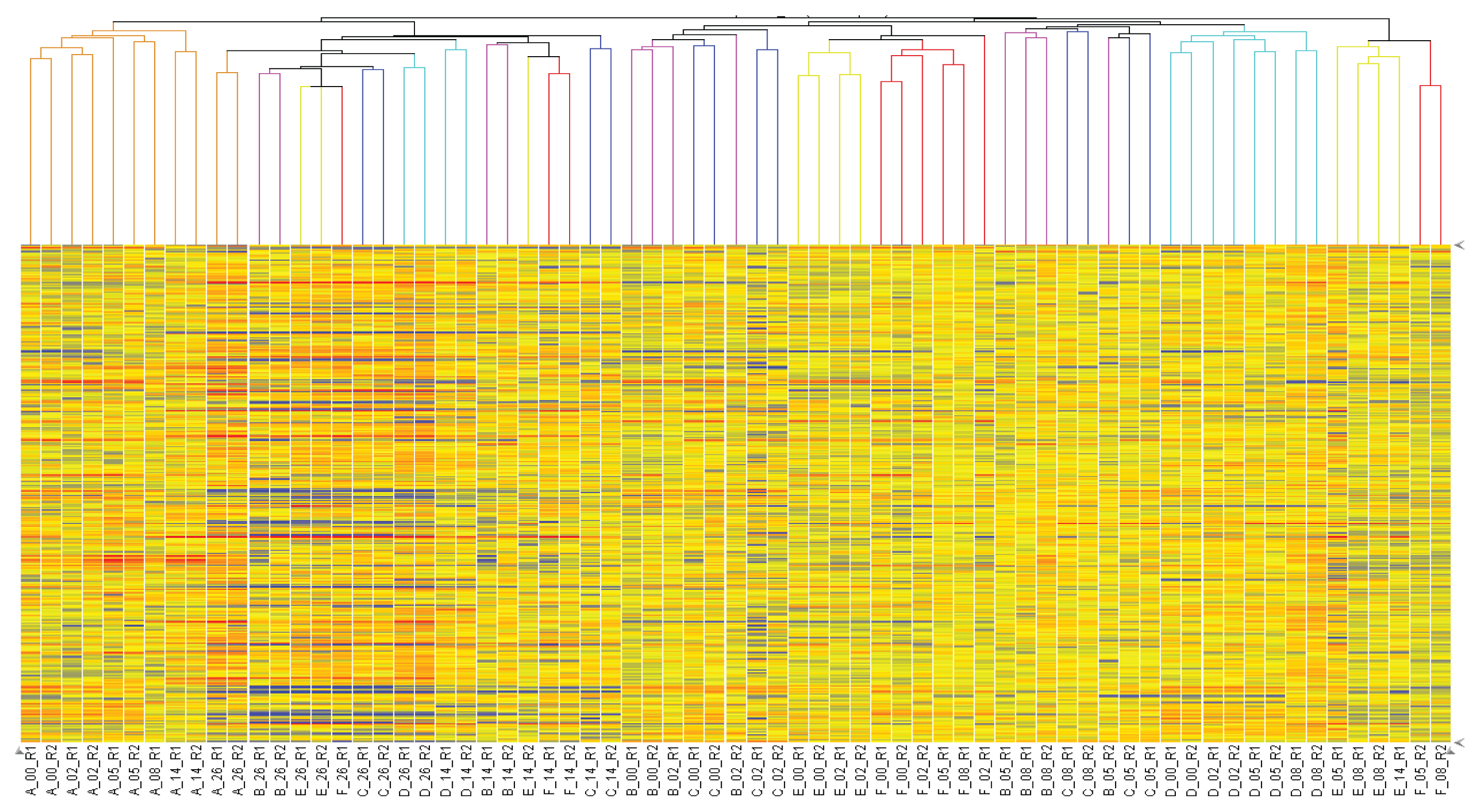

Supplement: Figure S1 — Hierarchical cluster analysis of 69 samples (RatTox U34 GeneChips). Heat map and dendrogram were obtained from 370 probe sets that were present or marginally present in all samples across all time points. Each column represents a sample (chip) and each row represents a gene. The replicate samples for each animal at each time points were clustered side-by-side, indicating a high level of technical consistency of microarray data. [file grsb-2007-235f4.tif]

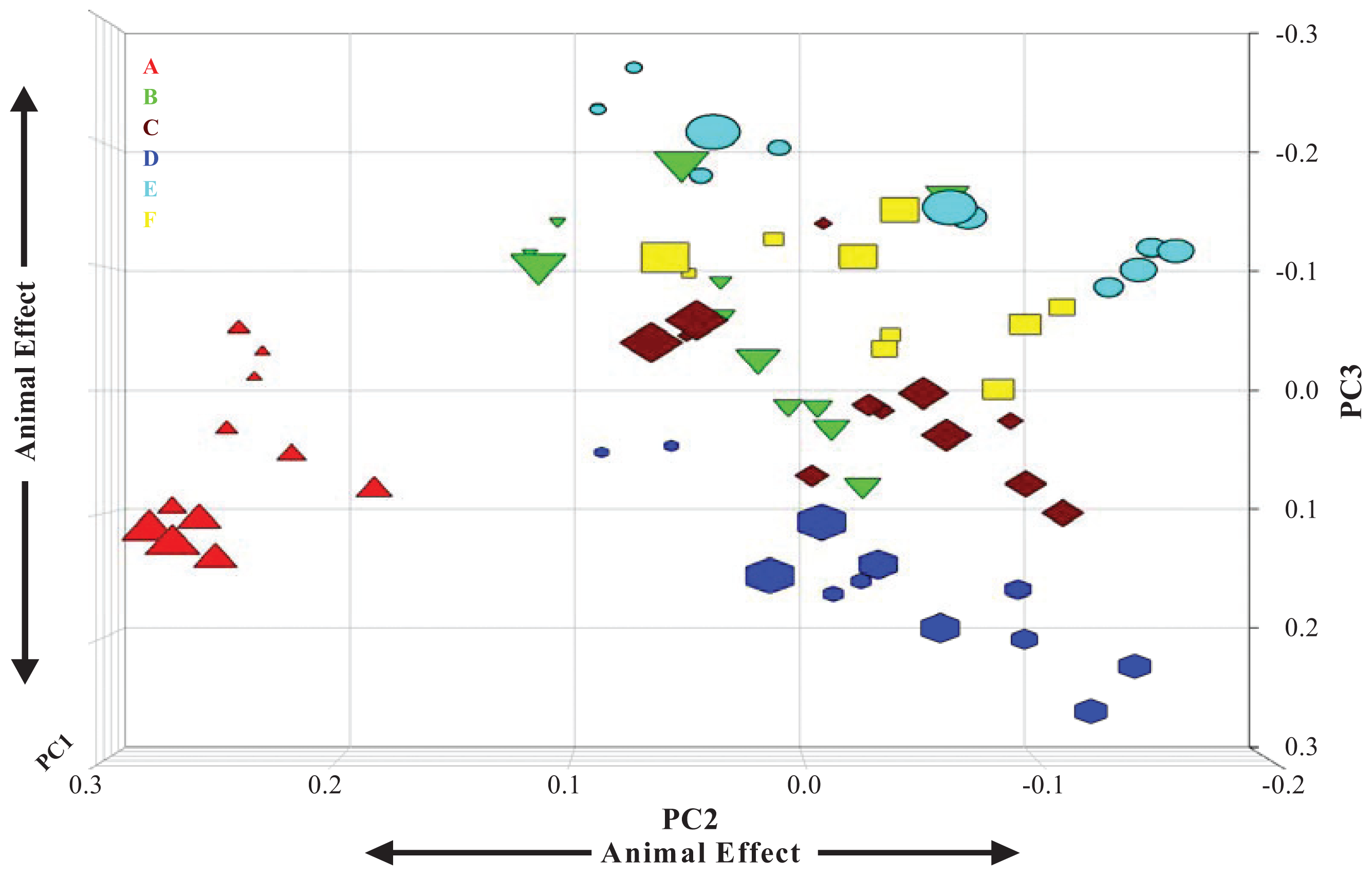

Supplement: Figure S2 — 3-D view of principal component analysis: PC1 – PC2 – PC3 projection. PCA was performed on 370 probe sets present or marginally present in all 6 animals. Time points are represented by different symbol sizes. The samples from different animals are represented in different colors: animal A in red, animal B in green, animal C in brown, animal D in blue, animal E in cyan and animal F in yellow. [file grsb-2007-235f5.tif]
